# Extended use or reuse of N95 respirators during COVID-19 pandemic: An overview of national regulatory authority recommendations

**DOI:** 10.1017/ice.2020.173

**Published:** 2020-04-22

**Authors:** Leticia Mitiko Kobayashi, Bianca Ramos Marins, Patrícia Cristina dos Santos Costa, Hugo Perazzo, Rodolfo Castro

**Affiliations:** 1 Fundação Oswaldo Cruz, Instituto Nacional de Controle de Qualidade em Saúde, Rio de Janeiro, RJ, Brazil; 2 Departamento de Saúde Coletiva, Universidade Federal do Estado do Rio de Janeiro, Rio de Janeiro, RJ, Brazil; 3 Departamento de Ciências Fisiológicas, Universidade Federal do Estado do Rio de Janeiro, Rio de Janeiro, RJ, Brazil; 4 Fundação Oswaldo Cruz, Instituto Nacional de Infectologia Evandro Chagas, Rio de Janeiro, RJ, Brazil; 5 Fundação Oswaldo Cruz, Escola Nacional de Saúde Pública Sergio Arouca, Rio de Janeiro, RJ, Brazil; 6 Universidade Federal do Estado do Rio de Janeiro, Instituto de Saúde Coletiva, Rio de Janeiro, RJ, Brazil

*To the Editor—*We read with great interest the article by Wang et al^1^ and the study by Cheng et al^2^ that highlighted the vital role of N95 respirators for preventing SARS-CoV-2 transmission and COVID-19 among healthcare workers (HCWs). The protective role of N95 respirators in other respiratory diseases could be translated to tackle the COVID-19 pandemic.^1^ Preliminary results in Hong Kong demonstrated that the use of N95 respirators for triage, for medical care of suspected or confirmed cases and during aerosol-generating procedures, drastically reduced COVID-19 infection among HCWs.^2^ We acknowledge that the effectiveness of N95 respirators to prevent SARS-CoV-2 transmission should be confirmed and their use in clinical practice should be supported during the COVID-19 pandemic. However, we are facing a scenario of global shortage in availability of personal protective equipment (PPE), including surgical masks and N95 respirators.^3^ Countries are in dispute over the insufficient number of manufacturers. With unfair markets and increasing prices, low- and middle-income countries are at risk of losing their ability to acquire PPE for their HCWs. Several studies have previously reported methods for PPE decontamination^4^ or reuse of N95 respirators.^5^

Globally, the discussion by health authorities regarding new approaches to managing the N95 respirator shortage is urgent. The extended use or reuse and/or implementation of decontamination methods of N95 respirators might be an alternative that can prevent SARS-CoV-2 transmission among HCWs during the COVID-19 pandemic. Therefore, we have summarized recommendations regarding the extended use or reuse of N95 respirators, and we provide an overview of published information by regulatory authorities, surveillance organizations, and ministries of health of several countries.

Two researchers independently scrutinized the websites of the regulatory authorities of countries or regions and of ministries of health that a members or associates of the International Coalition of Medicines Regulatory Authorities (ICMRA).^6^ Following the screening of information up to April 10, 2020, information from each country or region was collected in an electronic database. We collected the date of publication and information and excerpts from the guidance document regarding the recommendations for extended and reuse of N95 masks or filtering face pieces (FFPs). Extended use was defined as use for longer periods without removing the respirator (eg, treating several patients or working for >1 shift without interruption), and reuse indicated that the respirator was removed, stored, and used at least 1 more time.^7^

Overall, 27 countries or regions were screened: 5 countries (19%) only allowed extended use (Canada, France, Mexico, New Zealand and Sweden); 2 countries (7%) mentioned only reuse (Germany and Netherlands); and 3 countries (11%) recommended both strategies for rationing N95 respirators (Brazil, European region and the United States). No information was available on extended use or reuse of N95 respirators in the websites of 17 countries (63%). Some countries (Germany and Netherlands) recommended specific methods for N95 respirators decontamination, and others (Europe and United States) mentioned several options, leaving the decision to health services managers (Table 1). The following decontamination methods were mentioned: dry heat in a drying cabinet at 65–70°C (Germany); vaporous hydrogen peroxide (Netherlands, Europe, and the United States); ultraviolet germicidal irradiation and moist heat (Europe and the United States). The maximum duration of extended used ranged from 4 hours (France, New Zealand, and Sweden) to 40 hours (Mexico), and the maximum number of cycles of decontamination ranged from 2 (Germany) to 5 (United States).

**Table 1.** Recommendations for Extended Use or Reuse of N95 Respirators Among Health Professionals During COVID-19 Pandemic by Country and Regulatory Authority, 2020

Country and Regulatory Authority^a^	Extended Use	Reuse	Excerpted Summary Recommended Points on N95 Respirators	Update^b^
Brazil: National Health Surveillance Agency (ANVISA)	Yes	Yes	Allows the reuse by the same professional and extended use Recommends protecting the N95 respirators with face shield Infection Control Commission should discuss a local guidance Warns that the use for extended periods might degrade the mask	March 31, 2020
Canada: Health Products and Food Branch, Health Canada (HPFB-HC)	Yes	No	Allows use of masks after the expired deadline	April 9, 2020
Europe: European Commission Directorate-General for Health and Consumers (DG – SANCO) and the European Medicines Agency (EMA)	Yes	Yes	Prolonged used (4–6 h), if in right conditions and if not removed Reuse as a last-resort option to economize on the use of PPE Mentions the following possible methods for decontaminating and sterilizing masks (and other equipment) for reuse Steam, hydrogen peroxide vapor, ultraviolet germicidal irradiation and γ irradiation are under investigation, but none of the decontamination methods has been standardized.	March 31, 2020
France: French National Agency for Medicines and Health Products Safety (ANSM)	Yes	No	Allows prolonged use (up to 4 h) Switch in case of moisture or if integrity is compromised	NR
Germany: Paul-Ehrlich-Institute (PEI), Federal Ministry of Health and Federal Ministry of Labour and Social Affairs	Unclear	Yes	Allows reuse after heat treatment at 65–70°C in a drying cabinet for 30 min FFP2/3 masks from the United States, Canada, Australia, or Japan should be previously tested for resistance by a rapid temperature test at 70°C. All masks from Europe or China can be reprocessed. Must be personalized and can only be used by the same person Contaminated or defective masks must be disposed of immediately. Masks should be used up to a maximum of 2 decontaminations and then no longer used. Reprocessing measures will be limited in time (maximum of 6 mo) to build up national production capacities. The use of reusable respirators with interchangeable particle filters is an alternative meant to preserve resources.	March 31, 2020
Mexico: Federal Commission for the Protection against Sanitary Risks (COFEPRIS)	Yes	No	Recommends N95 respirators only for invasive and aerosol-generating procedures Allows prolonged use up to 40 h	NR
Netherlands: Medicines Evaluation Board (MEB)	Unclear	Yes	A short process with hydrogen peroxide gives an acceptable result both after visual inspection and based on the results of the test Hydrogen peroxide sterilizers are not available in all Dutch institutions The shelf life of reprocessed face masks should be determined	March 18, 2020
New Zealand: Medsafe, New Zealand Medicines and Medical Devices Safety	Yes	No	Up to 4 h of prolonged use for multiple patients with the same diagnosis	February 27, 2020
Sweden: Medical Products Agency (MPA)	Yes	No	Respiratory protection FFP2 and FFP3 are disposable, but if they are not removed, damaged or contaminated, they can be used up to 4 h according to WHO recommendations	April 2, 2020
United States: Food and Drug Administration (FDA) and Centers for Disease Control and Prevention (CDC),	Yes	Yes	The maximum length of continuous use in nondusty healthcare workplaces is typically dictated by hygienic concerns (eg, the respirator was discarded because it became contaminated) or practical considerations (eg, need to use the restroom, meal breaks, etc). No predetermined number of hours Use of a cleanable face shield Hang used respirators in a designated storage area or keep them in a clean, breathable container such as a paper bag between uses (so they do not touch each other) The person using the respirator should be identified. No more than 5 uses per device to ensure an adequate safety margin Vaporous hydrogen peroxide, ultraviolet germicidal irradiation, and moist heat are the most promising decontamination methods	March 27, 2020

Note. NR, not reported; FFP, filtering face piece.

a Countries, Regulatory Authority, for which was not possible to find specific recommendations related to N95 respirators: Australia, Therapeutic Goods Administration (TGA); Austria, Austrian Medicines and Medical Devices Agency (AGES MEA), Austrian Federal Office for Safety in Health Care; China, China Food and Drug Administration (CFDA); Denmark, Danish Medicines Agency; India, Ministry of Health and Family Welfare; Ireland, Health Product Regulatory Authority (HPRA); Italy, Italian Medicines Agency (AIFA); Japan, Pharmaceuticals and Medical Devices Agency (PMDA), and the Ministry of Health, Labour and Welfare (MHLW); Korea, Ministry of Food and Drug Safety (MFDS); Nigeria, National Agency for Food Drug Administration and Control (NAFDAC); Poland, The Office for Registration of Medicinal Products, Medical Devices and Biocidal Products (URPLWMiPB); Russia, Federal Service for Surveillance in Healthcare (Roszdravnadzor); Singapore, Health Sciences Authority Singapore (HSA); South Africa, Medicines Control Council (MCC); Spain, Spanish Agency of Medicines and Medical Devices (AEMPS); Switzerland, Swissmedic; United Kingdom, Medicines and Healthcare Products Regulatory Agency (MHRA).

b Update: date mentioned in the document.

Emergency use authorization (EUA) by the US Food and Drug Administration (FDA) allows the use of unapproved medical products or unapproved use of approved medical products. Currently, PPE items, in vitro diagnostic tests, and ventilators are included in the FDA EUA to tackle the COVID-19 pandemic. However, the FDA still does not allow sharing or reusing N95 respirators.^8^ Considering the COVID-19 pandemic specifically, the Centers for Disease Control and Prevention (CDC) published guidance regarding extended use and limited reuse of N95 respirators. Possible methods for decontamination cited as the most promising by the CDC were vaporous hydrogen peroxide, ultraviolet germicidal irradiation, and moist heat.^9^ In Brazil, the National Health Surveillance Agency (ANVISA) allowed the hospital infection control commissions (CCIHs) at each health service to create protocols for reuse by the same professional: use, withdrawal, packaging, assessment of integrity, time of use, and criteria for disposal.^10^

The impact of the COVID-19 pandemic in each country or region might be influenced by the number of cases, the proportion of patients needing hospitalization, and the infrastructure of healthcare systems. Health authorities should consider global PPE shortages and should define feasible recommendations for extended use or reuse or decontamination of N95 respirators. Regulatory agencies of few countries empowered health services managers to implement strategies for decontamination and/or reuse procedures. The Ministry of Labor and Social Affairs of Germany described the recommended decontamination method for N95 respirators in detail (ie, dry heat at 65–70°C in a drying cabinet for 30 minutes). On the other hand, up to 60% of the screened countries did not report any recommendations for extended use or reuse or decontamination of N95 respirators. In summary, we have provided some evidence that regulatory authorities are trending toward relaxing regulations during the PPE shortage. The extended use and reuse of N95 respirators have become the last resort because it is crucial to maintain HCW protection during the COVID-19 pandemic.

## Supplementary material

For supplementary material accompanying this paper visit http://dx.doi.org/10.1017/ice.2020.173. click here to view supplementary material

## References

[1] Wang Q , Yu C . The role of masks and respirator protection against SARS-CoV-2. Infect Control Hosp Epidemiol 2020 [Epub ahead of print]. doi: 10.1017/ice.2020.83. PMC7139364 32192550

[2] Cheng VCC , Wong SC , Chen JHK , et al. Escalating infection control response to the rapidly evolving epidemiology of the coronavirus disease 2019 (COVID-19) due to SARS-CoV-2 in Hong Kong. Infect Control Hosp Epidemiol 2020 [Epub ahead of print]. doi: 10.1017/ice.2020.58. PMC7137535 32131908

[3] Feng S , Shen C , Xia N , Song W , Fan M , Cowling BJ . Rational use of face masks in the COVID-19 pandemic. Lancet Respir Med 2020 Epub ahead of print]. doi: 10.1016/S2213-2600(20)30134-X. PMC7118603 32203710

[4] Lemmer K , Howaldt S , Heinrich R , et al. Test methods for estimating the efficacy of the fast-acting disinfectant peracetic acid on surfaces of personal protective equipment. J Appl Microbiol 2017;123:1168–1183. 28853204 10.1111/jam.13575

[5] Lin TH , Tang FC , Hung PC , Hua ZC , Lai CY . Relative survival of *Bacillus subtilis* spores loaded on filtering facepiece respirators after five decontamination methods. Indoor air 2018 May 31 [Epub ahead of print]. doi: 10.1111/ina.12475. PMC7165566 29855107

[6] International Coalition of Medicines Regulatory Authorities Membership Country/Region and Regulatory Authority websites. http://www.icmra.info/drupal/participatingRegulatoryAuthorities. Published 2017. Accessed April 10, 2020.

[7] Recommended guidance for extended use and limited reuse of N95 filtering facepiece respirators in healthcare settings. Centers for Disease Control and Prevention website. https://www.cdc.gov/niosh/topics/hcwcontrols/recommendedguidanceextuse.html. Updated March 2020. Accessed April 10, 2020.

[8] N95 respirators and surgical masks (face masks). US Food and Drug Administraion website. https://www.fda.gov/medical-devices/personal-protective-equipment-infection-control/n95-respirators-and-surgical-masks-face-masks. Updated April 2020. Accessed April 5, 2020.

[9] Decontamination and reuse of filtering facepiece respirators. Centers for Disease Control and Prevention website. https://www.cdc.gov/coronavirus/2019-ncov/hcp/ppe-strategy/decontamination-reuse-respirators.html. Updated April 2020. Accessed April 10, 2020.

[10] Guidelines for health services: prevention and control measures that should be adopted when assisting suspected or confirmed cases of infection with the new coronavirus (SARS-CoV-2) [in Portugese]. ANVISA website. http://portal.anvisa.gov.br/documents/33852/271858/Nota+T%C3%A9cnica+n+04-2020+GVIMS-GGTES-ANVISA-ATUALIZADA/ab598660-3de4-4f14-8e6f-b9341c196b28. Published March 30, 2020. Accessed March 31, 2020.

