# Supplementary material for: Extended use or reuse of N95 respirators during COVID-19 pandemic: An overview of national regulatory authority recommendations
Source: Infect Control Hosp Epidemiol. 2020 Apr 22:1–3. doi: 10.1017/ice.2020.173 (PMC7200848; doi:10.1017/ice.2020.173)
Supplement: Supplementary file 1 [file S0899823X20001737sup001.docx]

**Supplemental Material 1.** Countries/region members of the International Coalition of Medicines Regulatory Authorities recommendations regarding N95 respirators during COVID-19 pandemic, 2020.

| **Country: Regulatory Authority** | **Allows the prolonged use or reuse of N95 masks/respirators, regardless of manufacturer instructions (Yes or No)?** | **Extracted excerpts (when applicable, translated to English)** | **Summary recommended points on N95 respirators** | **Update (date of the document)** | **Link to Ministries of Health or Regulatory Authority’s website** |
| --- | --- | --- | --- | --- | --- |
| Australia: Therapeutic Goods Administration (TGA) | No | “Follow your healthcare facilities’ infection control policies around the use of masks and gowns.” | - Does not specify N95 masks, only discuss masks in general - It is not clear whether it allows the reuse or to use the mask for an extended time - It could be interpreted as a possibility of flexibilization of guidelines to allow local decisions | 23/03/2020 | <https://www.tga.gov.au/media-release/covid-19-advice-surgical-masks-and-gowns> |
| Austria: Austrian Medicines and Medical Devices Agency (AGES MEA), Austrian Federal Office for Safety in Health Care** | No | NA | NA | 09/04/2020 | <https://www.basg.gv.at/en/>  https://www.basg.gv.at/en/ |
| Brazil: National Health Surveillance Agency (ANVISA) | Yes   - Reuse - Prolonged use | “healthcare professionals should replace the surgical mask with an N95/PFF2 mask or equivalent when performing aerosol-generating procedures” “respiratory protection masks (N95/PFF2 or equivalent) may, in exceptionally be used for a longer period or for a number of times more than  as foreseen by the manufacturer,” “healthcare professional should use a face shield, as this equipment will protect the mask from contact with droplets expelled by the patient”  “protocol to guide health professionals, minimally, on the use, removal, packaging, evaluation of integrity, time of use and criteria for disposal of N95/PFF2 masks or equivalent (…) defined by the Infection Control Commission within healthcare units” "Masks worn for a longer period or for a number of times longer than provided for by the manufacturer may not meet the requirements for which they have been certificates. Over time, components such as strips and bridge material degrade, which may affect the quality of the fit and sealing." | - Allows the reuse by the same professional and for a longer period - Recommends protecting the N95 mask with face shield - Infection Control Commission should discuss a local guide - Warns that the use for long periods might degrade (could be interpreted as an allowance) | 31/03/2020 | <http://portal.anvisa.gov.br>  <http://portal.anvisa.gov.br/documents/33852/271858/Nota+T%C3%A9cnica+n+04-2020+GVIMS-GGTES-ANVISA/ab598660-3de4-4f14-8e6f-b9341c196b28> |
| Canada: Health Products and Food Branch, Health Canada (HPFB-HC) | Yes   - Prolonged use | “in times of increased demand and decreased supply, consideration can be made to use these expired N95 respirators. An expired mask can still be effective at protecting health care provider if: the straps are intact; there are no visible signs of damage; they can be fit-tested. Health care providers should inspect the mask and perform a seal check. There is no specific timeframe beyond the expiry dates for N95 respirators at which they would no longer be considered suitable for use.” | - Allows use of masks after the expired deadline. - An expired mask can still be effective in protecting the healthcare professional - It does not inform if it recommends storage and reuse. | 09/04/2020 | <https://www.canada.ca/en/health-canada/services/drugs-health-products/medical-devices/masks-respirators-covid19.html>  <https://www.canada.ca/en/health-canada.html> |
| China: China Food and Drug Administration (CFDA) | No | NA | NA | 12/03/2020 | <https://www.chinadaily.com.cn/pdf/2020/Guidelines.for.Personal.Protection.of.specific.groups.from.COVID-19.pdf>  <https://covid-19.chinadaily.com.cn/>  <http://english.nmpa.gov.cn/>    <https://www.cfdi.org.cn/cfdi/index_en> |
| Denmark: Danish Medicines Agency** | No | “For aerosol-producing procedures, such as the need for emergency airway management, staff should carry FFP2or 3 mask” | NA | 01/04/2020 | <https://laegemiddelstyrelsen.dk/en/> |
| Europe: European Commission - Directorate General for Health and Consumers (DG – SANCO) and European Medicines Agency (EMA) | Yes   - Reuse - Prolonged use | “In the event of shortages, to reduce consumption and maximise the use of PPE it is acceptable for staff to wear  the same respirator while caring for multiple patients with COVID-19 for up to 4–6 hours. This is on the condition  that the respirator is not removed between patients and is not damaged, soiled or contaminated, or unless specifically contraindicated by the manufacturer. Surgical masks are designed for single-use. Respirators are usually also discarded after use, but in the event of a shortage, they can be reused a limited number of times unless there is a risk of contamination through the deposition of infectious particles on the surface. Contamination of the surface of respirators and surgical masks entails a risk of infection when putting the device on again (donning) for reuse. Surgical masks are designed for single-use. Respirators are usually also discarded after use, but in the event of a shortage, they can be reused a limited number of times unless there is a risk of contamination through the deposition of infectious particles on the surface. Contamination of the surface of respirators and surgical masks entails a risk of infection when putting the device on again (donning) for reuse. Since SARS-CoV-2 survives in the environment, including on the surfaces of various materials such as tissue, there is a risk that the outer surface of respirators and surgical masks used during patient care, may become contaminated. The risk of the surface of surgical masks and respirators becoming contaminated by respiratory droplets is considered to be lower when they are covered with a visor. In such cases, reuse of the respirator/surgical mask may be considered as a last-resort option to economise on use of PPE. Research groups and healthcare facilities are currently looking into possible methods for decontaminating and sterilising masks (and other equipment) for reuse. Steam, hydrogen peroxide vapour, ultraviolet germicidal irradiation and gamma irradiation are being studied, but so far none of these methods have been standardised. Such options are only to be considered as an extraordinary last resort in the event of imminent shortages of PPE, depending on availability and feasibility after other approaches for the rational use of PPE (such as extended use) have been applied.” | - Prolonged used (4-6 hours), if in good conditions and if not removed - Reuse as a last-resort option to economise on the use of PPE - Mentions the following possible methods for decontaminating and sterilising masks (and other equipment) for reuse. Steam, hydrogen peroxide vapour, ultraviolet germicidal irradiation and gamma irradiation are being studied - so far none of these methods have been standardised. | 31/03/2020 | <https://www.ecdc.europa.eu/en/covid-19/preparedness-and-response>  <https://www.ecdc.europa.eu/sites/default/files/documents/Infection-prevention-control-for-the-care-of-patients-with-2019-nCoV-healthcare-settings_update-31-March-2020.pdf>  <https://ec.europa.eu/info/departments/health-and-food-safety_en>  <http://www.ema.europa.eu/ema/> |
| France: French National Agency for Medicines and Health Products Safety (ANSM) | Yes   - Prolonged use | “Allows prolonged use of a maximum of 4h with confirmed COVID-19 patients. If integrity is compromised, immediately switch.”  (Only in a video material) | - Allows prolonged use <= 4 hours - Switch in case of moisture or compromised integrity | NR | <https://solidarites-sante.gouv.fr/soins-et-maladies/maladies/maladies-infectieuses/coronavirus/professionnels-de-sante/article/covid-19-recommandations-de-protection-pour-les-personnels-de-sante>  <https://ansm.sante.fr/> |
| Germany: Paul-Ehrlich-Institute (PEI). Federal Ministry of Health and Federal Ministry of Labour and Social Affairs | Yes   - Reuse | “Use of protective masks in healthcare facilities. In view of the current supply bottlenecks for disposable medical nose protection masks (MNS) and filtering half masks (one-time FFP masks) all possibilities must be examined. In summary, this means that 3 categories that can be communicated accordingly: 1. MNS masks can be used at 65-70 degrees Celsius after appropriate reprocessing reused. 2. FFP2/3 masks with CE marking or those which are in accordance with the test principle of the central office of the countries for safety technology (ZLS), can be should also be reused after heat treatment. 3. FFP2/3 masks from the USA, Canada, Australia or Japan are reprocessing to undergo a rapid test of temperature resistance. Masks of Chinese origin are currently allowed to be the largest share of the volume. They fall into the number 2 and can be conditions mentioned above.  In detail: 1. Medical face masks, medical devices, standard DIN EN 14683  also mouth-nose protective masks (MNS masks) or colloquially "OP Masks" Objective: Protection of third parties (not the carrier) and thus primarily patient protection, patients may also be required to droplet infections compared to healthcare workers Avoid. These masks are used in large parts of the facilities of the  healthcare system. Suggestion: In the case of MNS masks, requirements in accordance with DIN EN 14683 be waived. All forms that provide protection can be used. before droplet transmission. Reuse of MNS masks without decontamination When used for external protection in everyday station life, in outpatient clinics or Care facilities can be reused. Prerequisite is a personalised commitment. When used in the operating room or in interventional. In principle, reuse is not possible. For reuse, there is a relationship between RKI and the Committee for Biological Substances (ABAS) coordinated process instructions. BMAS/BMG Template for the Crisis Staff of the Federal Government.  Decontamination of MNS masks before reuse: Decontamination of disposable-mouth-nose protective masks, e.g. Heat activation by dry heat at 65°C-70 °C for 30 minutes  recommended, other methods, e.g. at higher temperatures, can also be  MNS (see corresponding type check). 2. Breathing masks as personal protective equipment, standard DIN EN 149, which protects the carrier from Corona viruses by means of filter function (FFP 2 and FFP 3 masks)  Material resistance for decontamination masks with CE marking or equivalent standards  In the case of CE-marked masks, it must be assumed that the FFP2 and FFP3 masks are a treatment without changing the contour and material change exist, as part of the test. DIN EN 149 is a temperature conditioning of 70°C over 24 hours.  Masks without CE marking  Masks without CE marking scant view:  1. Masks designed in accordance with the test principle of the Central Office of the Länder for  safety technology (ZLS) have been approved as CE-marked  masks a temperature conditioning of 70°C over 24 hours  Learn. They can, therefore, be reprocessed.  2. Masks that are marketable in the USA, Canada, Australia or Japan,  are also considered to be marketable in DE. However, they will only be  conditioned at 38°C and must, therefore, be rapid test for temperature resistance at 70°C.  3. For arriving, non-CE-marked masks, a  further shortening of the entrance test according to ZLS (previously 5 days)  4For the possibility of reprocessing, it is necessary to temperature conditioning of 70°C, over 24 hours be provided.  Procedure instructions for decontamination by heat inactivation  SARS-CoV-2 by dry heat at 65°C-70 °C for 30 minutes for  the medical facilities  The following aspects should be taken into account in concrete terms and should be  hospital hygienists:  1. A procedure is set up in the institution to ensure that worn masks are in a safe way. Apparently contaminated or defective masks must be disposed of immediately. The institution shall ensure that the face masks can be stored without changing the quality of the masks or the decontamination process. It will strongly discouraged from wearing masks in a still-wet state closed containers, as this will lead to a short time massive reproduction of bacteria and moulds.  In particular, hygienic hand disinfection when putting on and off of the masks.  2. Masks must be personalised and, after decontamination, only by  to be used by the same person  3. The facility checks, at least visually and physically, that the masks not be affected by the process after decontamination  (shape and properties of the material).  4. Based on the previous data, the masks should be used up to a maximum of two  decontaminated and then no longer used.  5. To this end, the institution should set up a system indicating that a  mask has been decontaminated, and the number of  Decontamination steps per mask (e.g. by appropriate  marking on the mask).  6. The personnel who collect the collection (1st), review (4th) or decontamination, must be qualified and instructed to do so.  Be.  7. All procedural steps must be documented in such a way that verification is possible.  The reprocessing measures will be limited in time (max. 6 months, to build up own production capacities in DEU). During this time, increased the purchase of new masks and the process of  reprocessing continues to be validated.” | - Allows reuse after heat treatment, 65-70 degrees Celsius ºC) at the drying cabinet for 30 minutes - FFP2/3 masks from the USA, Canada, Australia or Japan should be previously tested for resistance by a rapid temperature test at 70 ° C - All masks from Europe or China can be reprocessed - Must be personalised and, after decontamination, only by to be used by the same person - Apparently contaminated or defective masks must be disposed of immediately - masks should be used up to a maximum of two decontaminated and then no longer used. - reprocessing measures will be limited in time (max. 6 months, to build up national production capacities - The use of reusable respirators with interchangeable particle filters is one another alternative to resource protection | 31/03/2020 | <https://www.bmas.de/SharedDocs/Downloads/DE/Thema-Arbeitsschutz/einsatz-schutzmasken-einrichtungen-gesundheitswesen.pdf?__blob=publicationFile>  <https://www.rki.de/DE/Content/InfAZ/N/Neuartiges_Coronavirus/Arbeitsschutz_Tab.html>  <https://www.pei.de/EN/home/home-node.html> |
| India: Ministry of Health  and Family Welfare | No | “Remove mask and throw into the trash” (video) | NA | 08/04/2020 | <https://www.mohfw.gov.in/> |
| Ireland: Health Product Regulatory Authority (HPRA) | No | “Surgical face masks should not be reused once removed e.g. when going to answer the telephone” | NA | 23/03/2020 | <https://www.hpsc.ie/a-z/respiratory/coronavirus/novelcoronavirus/guidance/infectionpreventionandcontrolguidance/ppe/Interim%20Guidance%20for%20use%20of%20PPE%20%20COVID%2019%20v1.0%2017_03_20.pdf>  <https://www.hpsc.ie/a-z/respiratory/coronavirus/novelcoronavirus/guidance/infectionpreventionandcontrolguidance/aerosolgeneratingprocedures/AGPs%20for%20confirmed%20or%20possible%20COVID19_v2.0_23032020.pdf>  <https://www.hpra.ie/> |
| Italy: Italian Medicines Agency (AIFA) | No | “It has been specified that FFP, in the current emerging and lack of such devices, must be made available, according to a priority criterion, to the highest professional risk operators manoeuvres and procedures that generate aerosols or operate in a context of prolonged high exposure to risk.”  “The indications contained in the document must find a local application, that declines them also taking into account the organisational contexts and the specific individual characteristics of  worker risk. In this regard, always considering the need to guarantee the availability of FFP for all operators who perform procedures capable of generating aerosols, the use of FFP can be assessed, in relation to the specific activities and services provided, the methods of work organisation and one overall and individual risk assessment; for example, in: organisational contexts where patients with COVID-19 infection are concentrated, especially when some of the patients undergo manoeuvres capable of generating aerosols, and the use of FFP can allow the operator to use the same PPE for a longer period of time; occasions when based on a careful risk assessment (individual characteristics  of the operator, structural characteristics of the environments), it is deemed necessary to adopt on the way  precautionary superior protection.  Finally, it is emphasised that the indications provided are interim, and could therefore be further and  promptly modified based on any new scientific evidence and changes in the  context conditions.” | NA | 01/04/2020 | <http://www.trovanorme.salute.gov.it/norme/renderNormsanPdf?anno=2020&codLeg=73770&parte=1%20&serie=null>  <http://www.trovanorme.salute.gov.it/norme/renderNormsanPdf?anno=2020&codLeg=73792&parte=1%20&serie=null>  <http://www.agenziafarmaco.gov.it/en> |
| Japan: Pharmaceuticals and Medical Devices Agency (PMDA), and the Ministry of Health, Labour and Welfare (MHLW) | No | NA | NA | 07/04/2020 | <https://www.niid.go.jp/niid/images/epi/corona/2019nCoV-01-200407.pdf>  <http://idsc.tokyo-eiken.go.jp/assets/diseases/respiratory/ncov/abstract.pdf>  <http://idsc.tokyo-eiken.go.jp/assets/diseases/respiratory/ncov/treatmentguid.pdf>  <https://www.pmda.go.jp/english/safety/info-services/safety-information/0001.html>  <https://www.mhlw.go.jp/english/>  <https://www.pmda.go.jp/english/> |
| Korea: Ministry of Food and Drug Safety (MFDS) | No | NA | NA | NR | <http://ncov.mohw.go.kr/upload/viewer/skin/doc.html?fn=1584078023495_20200313144023.hwp&rs=/upload/viewer/result/202004/>  <http://ncov.mohw.go.kr/upload/viewer/skin/doc.html?fn=1583390444687_20200305154046.hwp&rs=/upload/viewer/result/202004/>  [http://ncov.mohw.go.kr/duBoardList.do?brdId=2&brdGubun=24#](http://ncov.mohw.go.kr/duBoardList.do?brdId=2&brdGubun=24)  <https://www.mfds.go.kr/eng/index.do> |
| Mexico: Federal Commission for the Protection against Sanitary Risks (COFEPRIS) | Yes   - Prolonged use | “Depending on the indications in each centre, surgical masks may have a use of 8 continuous hrs, as well as the N95 respirators in conical form. The N95 shell-shaped respirators can be used for up to 40 hrs, as long as their structure is not altered (double, for example).”  “The procedures with recommendation of N95 use were taking oropharyngeal samples, invasive procedures, generation of aerosols, distance from patient of less than 1 meter.” | - Recommends N95 only for invasive and aerosol generating procedures - Allow the prolonged use for up to 40 hours | NR | <https://coronavirus.gob.mx/personal-de-salud/>  <https://drive.google.com/file/d/1WjSrb0O-TxQLDWng98AcXeKMklUcoUZp/view>  <http://cvoed.imss.gob.mx/wp-content/uploads/2020/02/Prevenci%C3%B3n_COVID-19.pdf.pdf>  https://www.gob.mx/cofepris |
| Netherlands: Medicines Evaluation Board (MEB) | Yes   - Reuse | “Use of 2 masks per cycle:  1-Process of 60°C per 12min with drying step, without detergent and chemical disinfection;  2-Process with drying step, without detergent, thermal disinfection at 90°C for 5 min;  3-Cleaning with drying step, with a cleaning agent (Strong MediClean) and thermal disinfection at 90°C for 5min;  4-Low pressure gas sterilisation of vaporised hydrogen peroxide and apply for 1, 2, 3, and 4 times.  5-Steam sterilisation at 134°C.  Masks that were not visually deformed were submitted for adjustment.  The preliminary result that once or twice sterilisation using the short process of hydrogen peroxide is acceptable, after a visual inspection and adjustment test.  Test limitations  • For each condition, only one adjustment test was performed  • Only one type of FFP2 face mask has been tested. Other available masks can, for example, contain cellulose. The presence of cellulose can be a limitation when using hydrogen peroxide. However, an initial exploratory study (without adjustment test) in The Martini hospital in Groningen did not indicate problems with the sterilisation of masks containing cellulose using hydrogen Peroxide.  • The treated masks were not worn or dirty. Dirt can negatively affect the effectiveness of the sterilisation process. It was not investigated to what extent the processes used can reduce dirt.  • Masks were individually packed in a laminated bag before sterilisation.  • The processes applied have not been validated for the treatment of  Masks.  • So far, a limited number of masks have been sterilised by  cycle (usually two, maximum four).  The efficacy of the treated masks has only been investigated with an adjustment test. No further studies have been conducted in relation to residues,  properties of the material or biological safety. No studies have been conducted  to determine whether the masks still meet ffp2 requirements.  Application in institutions  Based on the results of the exploratory research above, it appears that  it is possible to reprocess disposable FFP2 face masks. The following points should be taken into account:  • Reprocessing of potentially contaminated masks should not affect normal processes in the Central Sterilization Department  quality of other items that need to be sterilised. The aspects that should be considered are, for example:  - Whether or not masks should be packed before sterilisation;  - The place where the masks will be packaged (if applicable);  - Protective measures necessary for the handling of personnel masks;  • A process should be established in institutions to collect worn masks in a safe manner. The institution should pay attention to the  duration that masks can be stored without it negatively  affecting the quality of the masks or the reprocessing process.  • The institution should, at least visually and physically, inspect whether face masks are not affected by the process after reprocessing (shape and properties of the material).  • As the masks were individually packed during this study, no statements can be made about the effects of sterilisation of multiple face masks in one package.  • As moisture enters the mask during normal use, a drying  phase in the process of sterilisation of hydrogen peroxide will probably be necessary to prevent the premature termination of the sterilisation process due to the presence of moisture.  • A system must be configured to indicate that a mask has been  and, if possible, controls the number of reprocessing steps per mask.  • Hydrogen peroxide sterilisers are not available in all Dutch countries.  Institutions. Therefore, regional agreements should be made on use of this equipment.  • The shelf life of reprocessed face masks must be determined.” | - short process with hydrogen peroxide gives an acceptable result both after visual inspection and based on the results of the fit test - Hydrogen peroxide sterilisers are not available in all Dutch institutions - The shelf life of reprocessed face masks should be determined | 18/03/2020 | <https://www.rivm.nl/sites/default/files/2020-03/Hergebruik%20mondkapjes%20Informatie%20ENG_def.pdf>  <https://www.rivm.nl/en/documenten/reuse-of-ffp2-masks>  <https://english.cbg-meb.nl/> |
| New Zealand: Medsafe, New Zealand Medicines and Medical Devices Safety | Yes   - Prolonged use | “Respirators (e.g., N95, FFP2 or equivalent standard) have been used for an extended time during previous public health emergencies involving acute respiratory illness when PPE was in short supply. This refers to wearing the same respirator while caring for multiple patients who have the same diagnosis without removing it, and evidence indicates that respirators maintain their protection when used for extended periods. However, using one respirator for longer than 4 hours can lead to discomfort and should be avoided.” | - Cite a WHO document with recommendations - Up to 4 hours prolonged use for multiple patients with the same diagnosis | 27/02/2020 | <https://www.health.govt.nz/our-work/diseases-and-conditions/covid-19-novel-coronavirus/covid-19-novel-coronavirus-health-advice-general-public/covid-19-face-mask-and-hygiene-advice>  <https://apps.who.int/iris/bitstream/handle/10665/331215/WHO-2019-nCov-IPCPPE_use-2020.1-eng.pdf>  <https://www.medsafe.govt.nz/> |
| Nigeria: National Agency for Food Drug Administration and Control (NAFDAC) | No | “The respirator protects from the inhalation of droplets and particles. - Given that the fitting of different types of respirator will vary for each user, the respirator will require a fitting test in order to find the best match of PPE to  user. - A respirator should always be used when performing aerosol-generating procedures in a COVID-19 patient.” | NA | 14/03/2020 | <https://covid19.ncdc.gov.ng/resource/guideline/National%20COVID19%20Case%20Management%20Guide_NCDC.pdf>  <https://www.nafdac.gov.ng/> |
| Poland: The Office for Registration of Medicinal Products,  Medical Devices and Biocidal Products (URPLWMiPB)** | No | NA | NA | 20/03/2020 | <http://www.urpl.gov.pl/en> |
| Russia: Federal Service for Surveillance in Healthcare (Roszdravnadzor)** | No | NA | NA | NR | <https://www.roszdravnadzor.ru/en> |
| Singapore: Health Sciences Authority Singapore (HSA) | No | NA | NA | NR | <https://www.moh.gov.sg/covid-19>  <https://www.moh.gov.sg/covid-19/resources>  <https://www.hsa.gov.sg/> |
| South Africa: Medicines Control Council (MCC), Department of Health | No | NA | NA | 31/03/2020 | <https://sacoronavirus.co.za/category/tool-kits/>  <https://sacoronavirus.co.za/2020/03/31/covid-19-how-to-put-on-use-take-off-and-dispose-of-a-mask/>  <https://www.nrcs.org.za/news.asp?upd=1&newsID=4144>  <http://www.sanctr.gov.za/> |
| Spain: Spanish Agency of Medicines and Medical Devices (AEMPS)** | No | NA | NA | 09/04/2020 | <https://www.aemps.gob.es/>  https://www.aemps.gob.es/la-aemps/ultima-informacion-de-la-aemps-acerca-del-covid%E2%80%9119/ |
| Sweden: Medical Products Agency (MPA) | Yes   - Prolonged use | “Respiratory protection FFP2 and FFP3 are disposable, but if not removed, damaged or contaminated, they can be used up to 4 hours according to WHO recommendations.” | - Respiratory protection FFP2 and FFP3 are disposable, but if not removed, damaged or contaminated, they can be used up to 4 hours according to WHO recommendations. | 02/04/2020 | <https://www.lakemedelsverket.se/en>  <https://www.folkhalsomyndigheten.se/publicerat-material/publikationsarkiv/r/rekommendationer-for-handlaggning/?pub=70196> |
| Switzerland: Swissmedic | No | “Swiss Federal Council approved various measures designed to guarantee the supply of medical articles that are crucial for preventing and combating the coronavirus disease COVID-19” | NA | 03/04/2020 | <http://swissmedic.ch/swissmedic/en/home/medical-devices/market-surveillance-of-medical-devices/announcements-on-market-control-issues/inverkehrbringung_lebenswichtiger_beatmungsgeraete.html>  <https://www.admin.ch/gov/de/start/dokumentation/medienmitteilungen.msg-id-78686.html> |
| United Kingdom: Medicines and Healthcare Products Regulatory Agency (MHRA) | No | “The duration of use of PPE items should not exceed manufacturer instructions. Appropriateness of single versus sessional use is dependent on the nature of the task or activity being undertaken and the local context.” “Respirators are for single-use or single session use (section 6) and then are to be discarded as healthcare (clinical) waste (hand hygiene must always be performed after disposal) or if re-usable cleaned accorded manufacturer’s instructions. It is important that the respirator maintains its fit, function and remains tolerable for the user.” | - Reusable respirators should be cleaned according to the manufacturer’s instructions - Cleaning procedures are not clear | 10/04/2020 | <https://assets.publishing.service.gov.uk/government/uploads/system/uploads/attachment_data/file/878131/Frequently_asked_questions_on_wearing_PPE_v1.pdf>  <https://www.gov.uk/government/publications/wuhan-novel-coronavirus-infection-prevention-and-control/covid-19-personal-protective-equipment-ppe>  <https://www.gov.uk/government/organisations/medicines-and-healthcare-products-regulatory-agency> |
| United States: Food and Drug Administration (FDA) and Centers for Disease Control and Prevention (CDC) | Yes   - Prolonged use - Reuse | “During Contingency Capacity: extend the use of N95 respirators by wearing the same N95 for repeated close contact encounters with  several diferent patients, without removing the respirator (i.e., recommended guidance on implementation  of extended use)”  “During Crisis / Shortages: Implement limited reuse of N95 respirators by one HCP for multiple encounters with diferent patients, but  remove it after each encounter.”  “Definitions  Extended use refers to the practice of wearing the same N95 respirator for repeated close contact encounters with several patients, without removing the respirator between patient encounters. Extended use may be implemented when multiple patients are infected with the same respiratory pathogen and patients are placed together in dedicated waiting rooms or hospital wards. Extended use has been recommended as an option for conserving respirators during previous respiratory pathogen outbreaks and pandemics.(10, 11)”  “Reuse1 refers to the practice of using the same N95 respirator for multiple encounters with patients but removing it (‘doffing’) after each encounter. The respirator is stored in between encounters to be put on again (‘donned’) prior to the next encounter with a patient. For pathogens in which contact transmission (e.g., fomites) is not a concern, non-emergency reuse has been practiced for decades.(7) For example, for tuberculosis prevention, CDC recommends that a respirator classified as disposable can be reused by the same worker as long as it remains functional2 and is used in accordance with local infection control procedures.(9) Even when N95 respirator reuse is practised or recommended, restrictions are in place which limits the number of times the same FFR is reused. Thus, N95 respirator reuse is often referred to as “limited reuse”. Limited reuse has been recommended and widely used as an option for conserving respirators during previous respiratory pathogen outbreaks and pandemics.(2, 3, 10-12)”  “A key consideration for safe extended use is that the respirator must maintain its fit and function. Workers in other industries routinely use N95 respirators for several hours uninterrupted. Experience in these settings indicates that respirators can function within their design specifications for 8 hours of continuous or intermittent use. Some research studies (14, 15) have recruited healthcare workers as test subjects and many of those subjects have successfully worn an N95 respirator at work for several hours before they needed to remove them. Thus, the maximum length of continuous use in non-dusty healthcare workplaces is typically dictated by hygienic concerns (e.g., the respirator was discarded because it became contaminated) or practical considerations (e.g., need to use the restroom, meal breaks, etc.), rather than a pre-determined number of hours.”  “If reuse of N95 respirators is permitted, respiratory protection program administrators should ensure adherence to administrative and engineering controls to limit potential N95 respirator surface contamination (e.g., use of barriers to prevent droplet spray contamination) and consider additional training and/or reminders (e.g., posters) for staff to reinforce the need to minimise unnecessary contact with the respirator surface, strict adherence to hand hygiene practices, and proper PPE donning and doffing technique, including physical inspection and performing a user seal check.(16) Healthcare facilities should develop clearly written procedures to advise staff to take the following steps to reduce contact transmission:  Discard N95 respirators following use during aerosol generating procedures.  Discard N95 respirators contaminated with blood, respiratory or nasal secretions, or other bodily fluids from patients.  Discard N95 respirators following close contact with any patient co-infected with an infectious disease requiring contact precautions.  Consider use of a cleanable face shield (preferred3) over an N95 respirator and/or other steps (e.g., masking patients, use of engineering controls), when feasible to reduce surface contamination of the respirator.  Hang used respirators in a designated storage area or keep them in a clean, breathable container such as a paper bag between uses. To minimise potential cross-contamination, store respirators so that they do not touch each other and the person using the respirator is clearly identified. Storage containers should be disposed of or cleaned regularly.  Clean hands with soap and water or an alcohol-based hand sanitizer before and after touching or adjusting the respirator (if necessary for comfort or to maintain fit).  Avoid touching the inside of the respirator. If inadvertent contact is made with the inside of the respirator, discard the respirator and perform hand hygiene as described above.  Use a pair of clean (non-sterile) gloves when donning a used N95 respirator and performing a user seal check. Discard gloves after the N95 respirator is donned and any adjustments are made to ensure the respirator is sitting comfortably on your face with a good seal.  To reduce the chances of decreased protection caused by a loss of respirator functionality, respiratory protection program managers should consult with the respirator manufacturer regarding the maximum number of donnings or uses they recommend for the N95 respirator model(s) used in that facility. If no manufacturer guidance is available, preliminary data(19, 20) suggests limiting the number of reuses to no more than five uses per device to ensure an adequate safety margin.”  “Vaporous hydrogen peroxide, ultraviolet germicidal irradiation, and moist heat are the most promising decontamination methods. If FFR decontamination is considered, these methods do not appear to break down filtration or compromise the FFR; however, many of these methods can only be used for limited times.” | - the maximum length of continuous use in non-dusty healthcare workplaces is typically dictated by hygienic concerns (e.g., the respirator was discarded because it became contaminated) or practical considerations (e.g., need to use the restroom, meal breaks, etc.), rather than a pre-determined number of hours. - use of a cleanable face shield - hang used respirators in a designated storage area or keep them in a clean, breathable container such as a paper bag between uses - store respirators so that they do not touch each other - the person using the respirator is clearly identified - no more than five uses per device to ensure an adequate safety margin - Vaporous hydrogen peroxide, ultraviolet germicidal irradiation, and moist heat are the most promising decontamination methods | 27/03/2020 | <https://www.cdc.gov/coronavirus/2019-ncov/novel-coronavirus-2019-SupplyChecklist_of-N95-Respirators_COVID-19_4_6_20_num.pdf>  <https://www.cdc.gov/niosh/topics/hcwcontrols/recommendedguidanceextuse.html>  <https://www.cdc.gov/coronavirus/2019-ncov/hcp/respirators-strategy/index.html>  <https://www.cdc.gov/coronavirus/2019-ncov/hcp/ppe-strategy/decontamination-reuse-respirators.html>  <https://www.fda.gov/> |

** Associate members of International Coalition of Medicines Regulatory Authorities (ICMRA). NR: not reported; NA: not available
